# Lessons Learned From Beta-Testing a Facebook Group Prototype to Promote Treatment Use in the “Connecting Alaska Native People to Quit Smoking” (CAN Quit) Study

**DOI:** 10.2196/28704

**Published:** 2022-02-17

**Authors:** Pamela S Sinicrope, Colleen D Young, Ken Resnicow, Zoe T Merritt, Clara R McConnell, Christine A Hughes, Kathryn R Koller, Martha J Bock, Paul A Decker, Christie A Flanagan, Crystal D Meade, Timothy K Thomas, Judith J Prochaska, Christi A Patten

**Affiliations:** 1 Behavioral Health Research Program Department of Psychiatry and Psychology Mayo Clinic Rochester, MN United States; 2 Division of Consumer Communications Social and Digital Innovation Mayo Clinic Connect Rochester, MN United States; 3 Department of Health Behavior and Health Education School of Public Health University of Michigan Ann Arbor, MI United States; 4 Clinical & Research Services Division of Community Health Services Alaska Native Tribal Health Consortium Anchorage, AK United States; 5 Wellness and Prevention Division of Community Health Services Alaska Native Tribal Health Consortium Anchorage, AK United States; 6 Stanford Prevention Research Center Department of Medicine Stanford University Stanford, CA United States

**Keywords:** Web 2.0, social media, Facebook, Alaska Native, American Indian, Alaska, smoking, cessation, cancer prevention, Quitline, mobile phone

## Abstract

Social media provides an effective tool to reach, engage, and connect smokers in cessation efforts. Our team developed a Facebook group, CAN Quit (Connecting Alaska Native People to Quit smoking), to promote use of evidence-based smoking cessation resources for Alaska Native people living in Alaska, which are underused despite their effectiveness. Often separated by geography and climate, Alaska Native people prefer group-based approaches for tobacco cessation that support their culture and values. Such preferences make Alaska Native people candidates for social media–based interventions that promote connection. This viewpoint discusses the steps involved and lessons learned in building and beta-testing our Facebook group prototype, which will then be evaluated in a pilot randomized controlled trial. We describe the process of training moderators to facilitate group engagement and foster community, and we describe how we developed and tested our intervention prototype and Facebook group. All parts of the prototype were designed to facilitate use of evidence-based cessation treatments. We include recommendations for best practices with the hope that lessons learned from the CAN Quit prototype could provide a model for others to create similar platforms that benefit Alaska Native and American Indian people in the context of smoking cessation.

## Introduction

### Background

Social media is a powerful tool for reaching, engaging, and connecting smokers in cessation efforts. This could be especially true for Alaska Native people, who have the highest smoking prevalence in any US racial or ethnic subgroup [1]. Furthermore, Alaska Native residents of Alaska have a smoking prevalence more than double than that of Alaskan White residents (42% vs 17%), [2,3] but are difficult to reach with traditional face-to-face interventions because of their remote geography [4]. More than half of Alaska Native people live in more than 200 small communities (populations averaging 500-1000 total residents). Most of these communities are located off the road system and are accessible year-round only by a small plane, making them both geographically and socially remote.

Research indicates that internet (web)-based interventions are effective for smoking cessation and overcoming barriers related to travel; however, they are also associated with low use [5,6]. Conversely, social media has been shown to promote access and engagement among underserved, diverse populations with evidence-based content and peer support [7-9]. In contrast to individual-based treatments, social media platforms could lead to greater adoption and sustainability by encouraging collaborative efforts across adult generations that resonate with the Alaska Native cultural value of interdependence, defined as relationship oriented and collaborative in decision-making and lifestyle changes [10-12]. It is also important to note that traditional Alaska Native lifestyles, cultures, and holistic worldviews are intertwined and extend beyond mere social collectivism to include the interconnectedness of people with nature, the environment, and all elements of the universe [13,14]. Furthermore, Facebook is the dominant social networking platform used by 68% of US adults, more than double the proportion on Twitter (21%), Instagram (28%), Pinterest (26%), and LinkedIn (25%). Of Facebook users, 75% engage with the site daily. Facebook use indicates similarly high rates of engagement by sex (75% of females and 83% of males use Facebook), income (84% engagement among those earning <US $30,000/year vs 77% for those reporting >US $74,000/year), and age groups (88%, 18-29 years; 84%, 30-49 years; and 72%, 50-74 years), except for slightly less use (62%) among those aged ≥65 years. Of Alaskans, 91% reported having mobile broadband internet access [15-19], and surveys showed that the Alaska Native people in both rural and urban areas of Alaska use their smartphones to access social media, specifically Facebook [17,18,20].

On the basis of the above reasons, Alaska Native people, who value connection and community and represent an underserved health disparities group with significant barriers to receiving cessation treatment [21], may be optimal candidates for a social media platform where they can learn about evidence-based cessation resources in a group setting with other Alaska Native people also interested in quitting smoking. Furthermore, a Facebook intervention represents a potential platform for study recruitment, as Facebook is a primary source for social networking [17,18].

### Objective

The overall purpose of the CAN Quit (Connecting Alaska Native People to Quit Smoking) intervention study is to promote use of evidence-based smoking cessation resources available to Alaska Native people state-wide using social media. In the first 2 phases, we evaluated content for inclusion in the study using qualitative and quantitative methods [22,23]. In this third phase, our goal is to develop and beta-test the intervention prototype. The purpose of this paper is to highlight the results of the beta-testing phase of this 4-phase study and share lessons learned that could be applied by others interested in similar health behavior interventions [22].

### What Is the CAN Quit Study?

CAN Quit [22] is an ongoing 4-phase study to iteratively develop and pilot-test a culturally tailored social media–delivered intervention (via a secret Facebook group) to promote evidence-based smoking treatment uptake and cessation among Alaska Native people who smoke. The need for CAN Quit arose from a long-term collaboration between the Alaska Native Tribal Health Consortium (ANTHC) and Mayo Clinic. The goal of CAN Quit was to use peer support via social media to develop a scalable and sustainable intervention that could enhance use and reach of existing and effective cessation services offered to Alaska Native people through ANTHC, other Tribal cessation resources, and resources available through the Alaska State Quitline. If effective, we anticipate that ANTHC could manage and sustain CAN Quit as part of its tobacco cessation program and be moderated by its tobacco cessation counselors.

CAN Quit uses a culturally based digital storytelling approach [24-27], with content adapted from the effective Centers for Disease Control and Prevention (CDC) Tips from Former Smokers campaign [27] and from the ANTHC library of digital stories. Digital stories previously created by Alaska Native people contained their voices, pictures, and stories about tobacco cessation. We incorporated cultural variance and surface or deep structure frameworks [28] to address the influence of culture in designing health messages. The Mayo Clinic and Alaska Area institutional review boards and ANTHC Board of Directors’ Health Research Review Committee reviewed and approved all study phases.

In the first 2 study phases (described in the studies by Sinicrope at al [22] and Merculieff et al [23]), we gathered feedback first qualitatively and then quantitatively from Alaska Native people who smoke recruited state-wide primarily through targeted paid Facebook ads. We asked about the suitability of content, including digital stories and photos with text provided by CDC Tips and ANTHC. Similar to Tips, taglines to all content included a call to action for tobacco treatment by providing the (1) State of Alaska toll-free Quitline number, (2) URL to regional Tribal tobacco cessation program websites, and (3) URL to the smokefree.gov quit smoking resources website. In phases 1 and 2, through analysis of qualitative interviews with Alaska Native people who smoke and stakeholders (Alaska Native tobacco counselors) who viewed existing text, image, and video content, we found that participants preferred content that included Alaska Native people engaged in Alaska Native activities and were motivated to change smoking behavior when presented with images reflecting Alaska Native values of family, children, and community. Participants also preferred direct and honest storytelling styles told by other Alaska Native people without “sugar-coating” [23].

## Prototype Components

### Overview

**Figure 1:**
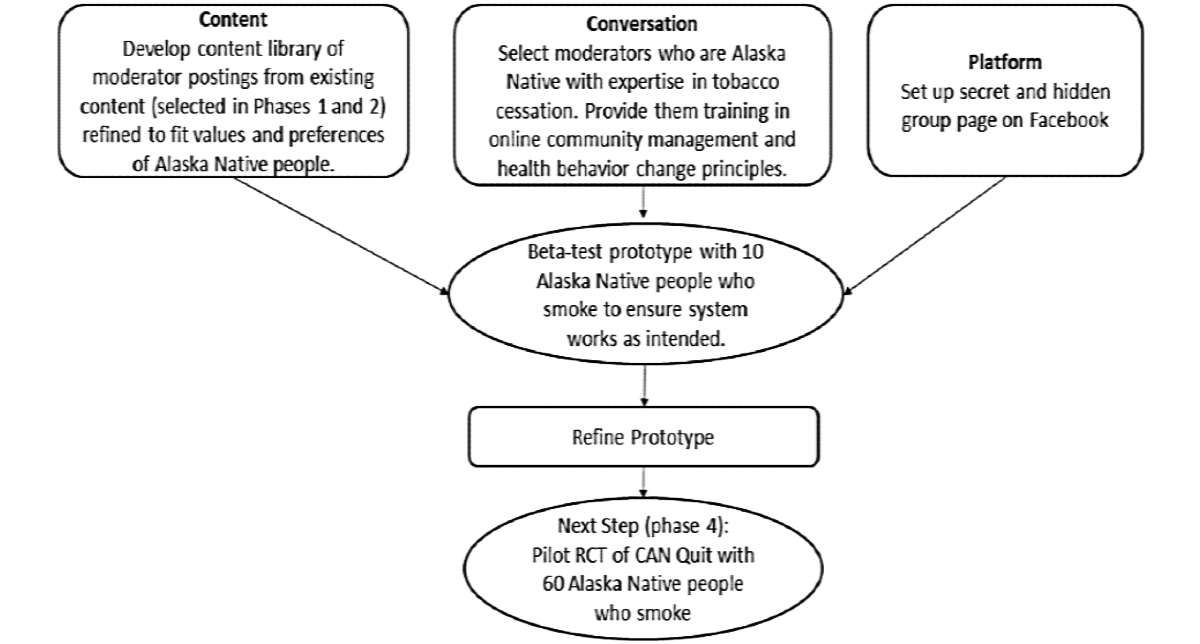
Overview of the CAN Quit (Connecting Alaska Native People to Quit Smoking) intervention prototype. CAN Quit is a 4-phase study. This figure highlights how phase 3 fits into the overall study design. RCT: randomized controlled trial.

Through this formative work, we developed an intervention prototype (phase 3) that included the following: (1) a Facebook group interface populated with culturally appropriate images and text outlining the purpose of the group, (2) a content library of images with text and digital stories (video) selected with sample moderator postings, and (3) a training plan for 2 Alaska Native study coordinators to moderate the Facebook group. Guidelines from Pagoto et al [29] informed the development of the components of our prototype and the training plan. Figure 1 provides an overview of the intervention prototype.

### Facebook Group Interface

We created community guidelines specific to CAN Quit to establish and maintain a supportive, inclusive, and respectful community. These were built on standard group conduct guidelines provided by Facebook when creating a group on the platform. The study team reviewed the standard group conduct guidelines together and elaborated on them to be specific to the CAN Quit group. For example, the group elements were as follows: (1) a name and cover photo for the group; (2) a description of the group’s purpose, including contact information and a link to evidence-based quitting resources; and (3) an *about* section describing the group, its purpose, and five basic guidelines: be respectful and courteous, no hate speech or bullying, protect yourself and the group’s privacy, no commercial posts or promotions and spam, and be careful about giving out medical advice. Privacy settings were established as *closed* (ie, only members could see who was in the group and what they post) and *secret* (ie, only members could find this group). Similar to CDC Tips content, prominent links to evidence-based quit smoking resources were located on all content and on the cover page.

### Content Library

The team developed a content library organized similar to a conventional treatment manual, available only to the moderators; however, once posted, Facebook stored the content on the page history and in the photo library. We organized pivotal discussion points by content, taglines, and sample text so that moderators could initiate discussions to promote quitting. The final 64-page library (digital and written) included the following: (1) welcome posts, group description, guidelines, and contact information; (2) options for Facebook cover photos; (3) 64 posts (images and text or video that included a tag line to call the Quitline or use Tribal quitting resources, similar to the CDC Tips campaign); (4) guidelines for handling inappropriate or misinformation (eg, deleting unrelated or false posts and removing or blocking participants breaking group guidelines); and (5) a list of quitting resources, including the State of Alaska Quitline and regional Tribal resources.

**Figure 2:**
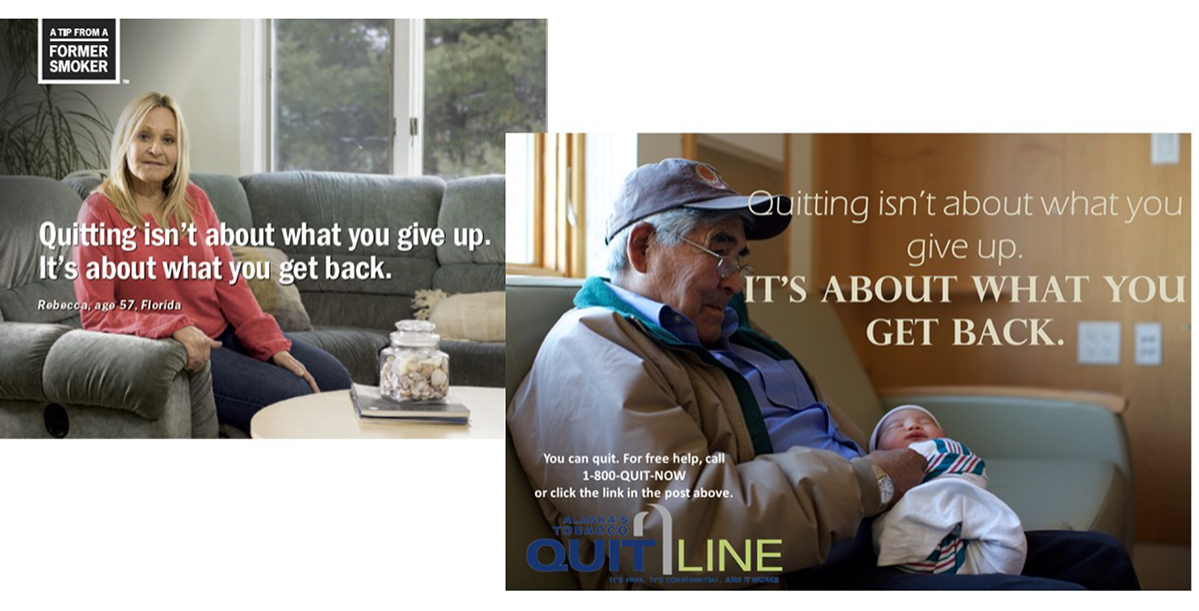
Example of Centers for Disease Control and Prevention Tips post and how it was refined for CAN Quit (Connecting Alaska Native People to Quit Smoking).

We organized posts by subject (ie, quitting reasons and sources of quitting support), and all content included sample text the moderators can modify to fit with or be responsive to current group conversations. Figure 2 includes 2 images, one is the original CDC post that features Rebecca, a 57-year-old White woman sitting on the sofa in her living room with the tag line “Quitting isn’t about what you give up. It’s about what you get back.” The second adapted image includes the same tag line, but the image is that of an Alaska Native grandfather holding a baby, which resonates with the Alaska Native value of family and its importance as a motivator for smoking cessation. Sample text in the form of the question, “What are you hoping to gain from becoming smoke-free?” accompanied the post in which the moderator could add in to generate a discussion.

### Moderators

The success of an online community is heavily dependent on the skills of moderators. Embedded in this skill is their familiarity with the topic (tobacco cessation), library content, understanding of and appreciation for Alaska Native cultural values, and ability to facilitate communication in a Facebook group. Recognizing this, we stressed hands-on scenario-based training and working directly with participants in each phase of the study so that moderators could continually develop their skills and become comfortable with working in the online community. Both Facebook moderators are trained tobacco specialists (TTSs) and Alaska Native employees of the ANTHC Tobacco Cessation Program in Anchorage, Alaska. Each received TTS training in motivational interviewing (MI) and additional training (as described in the section *Training Plan* below) to moderate CAN Quit. Moderators posted content every 2 to 3 days to prompt and reinforce participant posts and discussions (eg, sharing stories of tobacco use and quitting). Although content was prepared in advance in anticipation of discussions that would emerge, moderators also posted when relevant to guide and direct conversation and to provide timely content in response to member directions and needs.

## Training Plan

### Overview

A platform such as Facebook provides space for creating groups, but building a successful online community where members actively participate, engage with one another, and develop relationships requires an enabler (ie, a moderator) with organized strategic community management skills [30-33]. In addition, creating an online [30] community that serves Alaska Native people also requires that community managers and content developers pay attention to cultural norms and ways of interacting [31]. Therefore, the research team prioritized a moderator training using a scenario-based approach [34], providing moderators with skills to promote engagement, communication, and health behavior change (ie, use of evidenced-based quitting resources), whereas moderators provided feedback on how scenarios could be consistent with Alaska Native culture. The overall goal was to optimize and tailor the training specific to the needs of the CAN Quit study using *hands-on* instruction with real-life situations or scenarios relevant to learners [35]. In this instance, we created a CAN Quit practice Facebook group with posts and mock situations for moderators to practice crafting responses.

Two phases of training for moderators covered essential elements of online community management and moderator practices in part 1 and MI, tailoring, and cultural adaptation in part 2. The moderators actively participated in developing the training, providing their expertise and background as Alaska Native TTSs working with Alaska Native people.

### Online Community Management and Moderator Practices Training

Over the course of 12 training hours, moderators acquired skills in strategic community management and applicable moderator practices required to build and maintain a thriving online health community (presented by CY). The training included the following: (1) a pretraining questionnaire; (2) a 90-minute web-based introduction to online community management (see the section *Online Training*); (3) a 2-day in-person workshop (10.5 hours) with hands-on practice moderating and time for feedback and cultural tailoring; and (4) ongoing coaching through observing moderation practices in situ.

#### Pretraining Questionnaire

The pretraining questionnaire asked moderators four questions: (1) What do you hope the CAN Quit Facebook group will achieve? (2) As a moderator, what do you anticipate will be your single biggest challenge? (3) As a moderator, what type of interaction in the community would make you feel rewarded? and (4) What does success look like? Answers to these questions helped tailor the initial web-based training session.

#### Online Training

This 90-minute training was divided into 2 sessions. Session 1 introduced the fundamentals of community management, such as defining online community and understanding why people join patient support networks and what motivates them to return. The session addressed the interplay among the three ingredients for success: growth, activity, and sense of community. It also explored strategies and tactics supporting these items throughout the life cycle of an online community. The session examined strategic approaches for program inception: (1) welcoming new members and integrating them into the community; (2) ensuring every post receives a response; (3) connecting members; (4) keeping people talking; (5) asking questions that encourage self-disclosure and build trust; and (6) discovering members’ knowledge, capacity, and strengths.

During the subsequent session, trainees posed questions to the community management expert or trainer in *Ask Me Anything* style. In this session, questions frequently include the following: (1) How do you engage inactive members? (2) How do you encourage meaningful interactions? (3) What do you do about members who do not follow community guidelines? Although these questions were addressed in brief during the *Ask Me Anything*, we also addressed them in the in-person workshop.

#### In-Person Workshop

**Figure 3:**
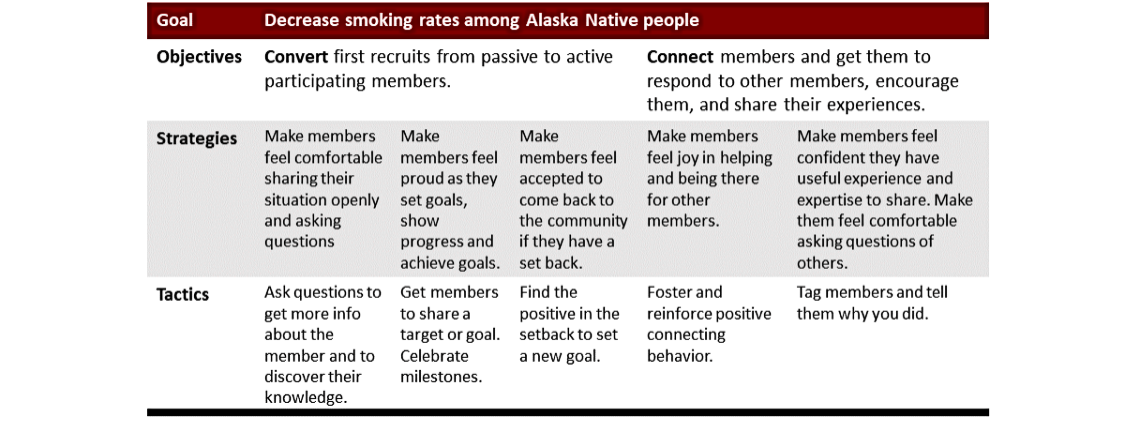
CAN Quit (Connecting Alaska Native People to Quit Smoking) strategic community plan.

A strategic community management plan ensures that the achievements of the online community and its members align with the overarching goal of sponsoring institutions. In this 2-day workshop, moderators and trainers developed a strategic community management plan (Figure 3), followed by hands-on practice applying strategies consistent with situational learning strategies [34]. The trainer (CY) relies on the best practices by Millington [30] adapted to a health setting in this study and in similar work with the online community Mayo Clinic Connect [32,36,37].

#### Strategic Planning Framework

The first day of the workshop focused on how to establish a framework for building an online community that includes the building blocks (goal, objectives, strategy, and tactics). The goal was defined as the direct value the organization gets from the community. Objectives are what members need to do to get this value. Strategy involves the emotion moderators amplify to get members to perform this behavior. Tactics are the things moderators do to amplify this emotion [30].

Importantly, the workshop emphasized that each action moderators take in the online community should be meaningful: (1) give new members confidence to make a first post, get help, and return to help others; (2) demonstrate the value of the CAN Quit group, a community where no question goes unanswered and people are supportive; and (3) amplify a sense of personal progress, success, and growing confidence in each member as they reach milestones and goals, or return for encouragement and support if a setback is experienced. Trainees were asked to reflect on the following questions: (1) What do you want members of the CAN Quit group to do? and (2) What do you have to do to make that happen? In the inception stage of this limited enrollment group, tactics were focused on moderator actions promoting activity and a sense of community objectives.

The remainder of the 2-day workshop concentrated on specific moderator approaches to support the above tactics. Although the moderator approaches remained platform agnostic, specific Facebook tools and their optimization were also discussed and a hidden *Practice CAN Quit* group was created to practice skills. Trainees learned and practiced the following: (1) *Setting up the group*: What do you want members to see when they visit the group? What 1 action do you want them to take? (2) *Getting the conversations started:* How do you create conversations members want to participate in? (3) *Managing the tough stuff:* How do you deal with members who do not follow the community guidelines? (4) *Effectively using private messaging:* When should you use and not use private messaging? and (5) *Managing the group when it becomes active:* When should a moderator stay out of the way? How do you transition from micro to macro interactions? The team spent additional time discussing ways to monitor or track posts, strategy, and tactics to determine which may need adjustment.

The training wrapped up with a reminder of the importance of self-care. From the experience of the trainer (CY), reading all posts, responding with empathy, and helping members succeed can be emotionally taxing. The group addressed good self-care practices, including recognizing when to unplug from the group and taking time off and reaching out to fellow moderators and the research team for difficult situations or decisions.

#### Ongoing Coaching Through Observing Moderation Practices In Situ

Following the training, trainer CY provided moderators feedback biweekly, as they put the newly acquired skills into practice. They were given tips on how posts and interactions could be tweaked and recognizing opportunities to take action to promote further engagement. Moderators also reached out to other members of the research team with questions and concerns.

### Effective Communication Strategies and Cultural Adaptation Training

In this training, moderators received approximately 10 hours of web-based training, divided across 2 days, approximately 5 months apart (July and December 2019). The trainer (KR) addressed the following: (1) theories of behavior change and tailored communications, (2) application of MI techniques for social media interventions, and (3) cultural adaptation of smoking cessation messages.

#### Theory of Behavior Change and Tailored Communications

The group discussed several behavior change conceptual models, including self-determination theory [38] and cognitive behavioral therapy [39,40]. The author KR also presented principles of cultural and psychosocial tailoring of health communications [28,41], at both the individual and group levels. He then applied theoretical models to cases directly addressing smoking cessation behavior in an Alaska Native population.

#### MI Techniques in a Social Media Intervention

MI is a client-centered counseling approach that has been tested in hundreds of studies, including numerous studies on smoking cessation [42]. This aspect of the training emphasized how to use key MI principles (eg, autonomy support) and strategies (rolling with resistance and eliciting change talk) of MI in the context of this social media intervention. The group discussed and practiced strategies for writing effective questions and reflective responses and for acknowledging and eliciting change talk, such as the following:

- “Sounds like you are moving toward giving up smoking.”
- “Your post indicates you have lots of good reasons to quit, such as your kids, your community, and your health.”
- “You are proud of yourself for staying quit for more than two weeks.”
- “Although you are not quite ready to quit yet, it sounds like you are moving in that direction. You are getting close.”

#### Cultural Adaptation of Smoking Cessation Messages

Moderators received training on incorporating the Alaska Native cultural content into their Facebook posts and responses. This included a review of Alaska Native core values and the practice of generating messages that linked smoking cessation to these values. For example, the team brainstormed ways that respect for nature, community, humility, and mindfulness could be linked to cessation messages. The team also explored ways to post polls on the Facebook page that queried users’ core values, which they could then use to generate tailored messages about how their values might drive their efforts to reduce their tobacco use.

Training applied web-based exercises and actual or simulated Facebook posts in a scenario-based learning approach to enable moderators to master concepts and build messaging skills [34]. When given posts, the moderators generated responses in real time using the principles and strategies presented. For example, moderators received the following participant post:

I know quitting is going to be hard but I want to show my kids that I’m strong and I want to be around for them when they’re older and have their own kids. I want to be there for my family.

For this case, the response included the strategy of rolling with resistance around the perceived difficulty of quitting and use of a reflective statement capturing the meaning of quitting for this participant; that is, change talk about her desire to be there for her family, such as using a reflective state, for example, “on one hand you are scared that quitting will be challenging,” as well as reflecting the meaning of quitting back to the participant that is her change talk, for example, “but quitting would send them a message that you care about them and are willing to do what’s needed to be there for them.”

## Beta Test

### Overview, Sample, and Methods

Upon completion of all training and 2 weeks of practice, we beta-tested the intervention prototype with our 2 moderators and a sample of 10 Alaska Native adults living in Alaska who smoke cigarettes. As in our prior study phases described previously [22], Alaska Native people who smoke were recruited state-wide primarily via targeted paid Facebook ads that included an image and short text consistent with Facebook’s advertising guidelines based on the following criteria: aged >19 years, self-reported Alaska Native race or ethnicity, and keywords related to tobacco use. To be eligible to participate, participants had to be living in Alaska, aged ≥19 years, have smoked >1 cigarette per day over the past 7 days with cigarettes as the main tobacco product used, and be considering or willing to make a quit attempt. They also had to have access to broadband (high-speed) internet on a mobile phone, at home, work, or other locations, and a Facebook account or be willing to set one up. Finally, they could not have been enrolled in a cessation program or using pharmacotherapy to stop smoking during the last 3 months. Prior work suggested 10 people as the minimum for optimal engagement in social media [43,44].

The purpose of this phase was to provide participants 30 days of exposure to the CAN Quit Facebook group and obtain their feedback to (1) ensure the system worked as intended, (2) identify technical issues, and (3) facilitate program refinements in preparation for the pilot randomized controlled trial (RCT). We administered a web-based survey at baseline to collect demographic, smoking behavior, and readiness to quit information. At completion of the 1-month beta test, we assessed readiness to quit, quit attempts, and use of cessation resources. In addition, we also assessed the acceptability of the CAN Quit group platform via the social media usability scale, which measures perceived ease of use and usefulness (3 items each), ease of learning (1 item), and satisfaction (6 items), rated on a 5-point scale (1—strongly disagree, 5—strongly agree) [45]. We also asked open-ended questions to provide feedback on modifications to the prototype. Participants received a US $25 gift card for their time after each assessment.

### Analyses

We summarized the sample characteristics and usability measures using descriptive statistics (means, SDs, percentages, and frequencies). We analyzed the responses to open-ended questions using content analysis [46].

### Results

#### Baseline

Of the 10 Alaska Native adults (mean age 35, SD 8.18; range 23-52 years; 9/10, 90% female; 7/10, 70% living in a rural area; 6/10, 60% employed for pay; and all had a high school degree or higher), the average number of cigarettes smoked per day was 10.2 (SD 7.5; range 2-25) at baseline. Furthermore, 60% (6/10) reported a high level of readiness to quit cigarettes (mean 7.2, SD 2.7; range 2-10), and 50% (5/10) reported readiness to use smoking cessation treatment (mean 7.1, SD 2.6; range 0-10) (data not shown).

#### One-Month Follow-Up

Among the 10 participants, 6 reported at least one quit attempt and 2 quit smoking (both called the Quitline). Feedback on the Facebook group revealed an overall social media usability score of 4.1 (SD 0.58; range 3.1-5.0) for the 13 items reported. Mean categorical scores were as follows: for usefulness (3 items), 3.7 (SD 0.66; range 3.0-5.0); for ease of use (3 items), 4.5 (SD 0.61; range 3.0-5.0); for ease of learning (1 item), 4.7 (SD 0.67; range 3.0-5.0); and for satisfaction (6 items), 4.0 (SD 0.71; range 3.1-5.0).

#### Facebook Page Activity Summary

We conducted a beta test in the fall of 2019. Moderators posted 19 unique posts from the content library, 1 participant created a post with a photo of their children as a reason for quitting, and the cover page was posted. Participants posted a total of 35 reactions (eg, all *likes* and *loves*), and 130 comments were generated across the posts, with 57.7% (75/130) of comments coming from moderators and 42.3% (55/130) from users. Of the 10 pilot testers, 9 posted 54 comments in all. The number of comments per person ranged from 1 to 24 (median 5). For the 2 people who reported quitting smoking, 1 posted 15 comments and the other posted 2. There was only 1 instance where a participant shared content outside the group on a personal Facebook page. When this occurred, the moderator sent the participant a private message, redirecting the participant to the group rules. There were no other boundary violation incidents (data not shown).

#### Participant Feedback on Group

Participants and moderators were asked to provide feedback on how to improve moderator posts as well as the Facebook group overall (Table 1). Four areas of response emerged: (1) providing incentives for participation, (2) clarifying the group purpose, (3) making posts more interactive and specific to rural Alaska Native people, and (4) encouraging group members to post more frequently. Moderators expressed that they felt prepared to use the skills from their training. They expressed challenges associated with working with the Facebook platform to ensure they did not miss comments because Facebook does not organize posts in chronological order in groups. They also expressed challenges associated with keeping track of multiple users across posts and found that maintaining a spreadsheet of group members and their stories helped to ensure that they responded appropriately to posts. One of the biggest challenges was responding to participants who shared traumatic or personal information. However, they expressed that training in MI principles of communication and self-care was especially helpful in this regard (Table 1 and Table 2).

**Table 1:** Moderator feedback on CAN Quit (Connecting Alaska Native People to Quit Smoking) beta test.

Question and moderator feedback		Changes made
**What were the most challenging parts of moderating the CAN Quit group?**		
	Maintaining communication among participants on multiple threads at one time. It can be easy to miss someone’s response if you are not diligent.	Study team monitors group and notifies moderators if they miss a post
	There were participants who shared personal experiences (eg, death of a child and struggles with other substances) with the group, and it was challenging to figure out how to best respond to these comments. There was also a participant who used our post to sell items on a personal FB^a^ page.	Consultation with the trainer (CY) before crafting a response Determine as a group the best way to handle more sensitive topics Added an additional refresher training in MI^b^ principles (KR) and practices before RCT^c^
	Tracking each participant’s responses so you can easily reference prior conversations. This is important when building rapport with the participant.	Moderators maintain a spreadsheet tracking all members with notes to help them remember details and respond accordingly
	Connecting one person to another based on their personal experiences and actually getting a response or any dialogue between the two.	Feedback or reinforcement from the trainer (CY) on different tactics for connecting participants (ie, tagging, private messaging, and asking questions specific to interests or strengths of participants).
**What advice would you give to someone who was about to moderate a group like CAN Quit?**		
	Keep a log of your communication with each participant from when the group starts. This way you can easily reference this spreadsheet to see what topics have been discussed with this participant. This can be especially helpful in groups with more than one moderator.	N/A^d^
	Be thoughtful in your responses; sometimes it can be difficult to not sound robotic over the computer. I think this is important in developing a connection with each participant and building trust. The MI tips we learned were very helpful in making sure we are professional but personable when moderating.	N/A
**What did you think you did best and what do you think you need to work on?**		
	We did a good job of posting new content with an interesting prompt every few days which helped the group stay active.	N/A
	We did a good job of refining our responses to each participant and knowing when to refer the Quitline. We do not want to refer the Quitline so often that we sound robotic but we want to encourage participants to use evidence-based smoking cessation programs.	Use MI skills training and created additional posts that discuss the Quitline and Tribal quitting resources
	We are working on generating quicker responses	Moderators share the responsibility of monitoring the Quitline

^a^FB: Facebook.

^b^MI: motivational interviewing.

^c^RCT: randomized controlled trial.

^d^N/A: not applicable.

**Table 2:** Beta tester feedback on CAN Quit (Connecting Alaska Native People to Quit Smoking) beta test.

Improvement areas (themes)	Representative quotes	Solutions implemented by team
Clarify group purpose	“Have mental or behavioral health programs ready” “I felt unsure if I was supposed to use the site to access the quit helpline through FB^a^, because my region doesn’t use the Quitline for assistance in quitting anymore”	Additional welcome posts created that clarify group purpose as well as the Quitline and Tribal Quitting resources Moderators incorporated feedback to reinforce FB page purpose and the quitting resources
Provide incentives or games	“It would be great to get the rural communities involved and offer some sort of Photo, Video or Art contest that included a drawing, a photo or video that helps people quit smoking or talk about the negative effects of smoking. It would draw more people in and get people to participate in the efforts toward Tobacco Prevention and keep them busy toward something positive. If we could include a prize for 1st, 2nd and 3rd also!” “Maybe some fun trivia games to get people wanting to look at the page.” “Do more instead of just post posters. I mean we all can brainstorm in a different way and play along as we go?”	Team implemented “polls” where participants could vote on what values are important to them and write in additional values Will add incentives for future groups
How to improve posts	“Could use less medical type photos. More of our own pictures to help support each other” “I think more should be put into it. More involvements” “More videos that include rural areas would be a great idea” “Posts being more clear about regional resources would be nice” “Make more posts”	More rural posts will be used in the randomized controlled trial More posts relating to Alaska Native values were created using Alaska Native imagery (eg, people, families, communities, and activities) New post and discussion prompt posted to the group every 2 days Combined posts and video links to increase participant engagement Creation of interactive posts like the FB poll Developed a moderator spreadsheet to track posts to ensure full variety of topics and types of posts (eg, urban, rural, photo or text, and video) will be addressed
Moderators make group more interactive	“Maybe encourage more people to post more often to gain connection.” “Find ways to make it more interactive.”	Moderators will tag participants to increase participation on individual posts Moderators individually welcome all new members on the welcome post and encourage them to interact. Prompted new members to introduce themselves and their reasons for wanting to quit. Moderators connect members to interact who express similar experiences or concerns Provided additional training to moderators that focused on health communication and ways of increasing interaction Developed follow-up questions that may be asked on the original post to encourage discussion

^a^FB: Facebook.

## Conclusions, Discussion, and Overall Recommendations

This paper shares the processes involved with developing and beta-testing a social media group as an intervention to promote use of evidence-based resources for quitting smoking using peer support. We share specific approaches, strategies, and methods for stimulating participant engagement in the Facebook group and emphasize the importance of moderator training and creating a content library. During training, trainers and moderators shared expertise to help build cultural competency and web-based communication skills that would build community and promote behavior change. Cultural tailoring is a critical component of the prototype. The research team used content linked to core Alaska Native values and/or Alaska Native imagery when possible, and Alaska Native moderators used their personal experience to incorporate cultural values and content into their written posts and responses. The purpose of these phases was to adapt the intervention and prepare moderators to deliver the intervention for the subsequent phase, which was a pilot RCT.

The results of the beta test were encouraging with participants expressing a high degree of usability of the CAN Quit Facebook group based on the Social Media Usability Measure, and there was a signal of potential effectiveness, with 2 out of 10 participants using the Quitline and reporting quitting smoking. Qualitative feedback was helpful in directing the research team to specific improvements needed to make the Facebook group more successful or usable; for example, providing additional training to moderators to promote interactions among participants, support conversations that may contain emotional content, and add more welcome posts to the group page to better clarify the group’s purpose: use of evidence-based quitting resources. Given that the group is on Facebook, which is popular in Alaska Native communities [16], most users were not new to the technology, which made it easier to avoid technical challenges in participating. We noticed that no video content was posted by moderators and some other content was posted repeatedly, whereas other content was not used. The selection of content was based on the activity of their group members at the discretion of the moderators. However, based on this observation, we determined that it would be helpful to the moderators as they select content, to develop a spreadsheet to track posts already used from the content library.

Strategies to promote engagement between participants rather than between participants and the moderator are the primary areas for improvement. Strategies suggested by our community management expert (CY) included tagging, private messaging, pinning posts so they show up at the top of the group feed, and drafting new follow-up questions to add to posts to further stimulate discussion. Several strengths were noted during the beta-testing phase. Moderators were able to model empathy and support, which included their adept use of reflective listening skills and personal experiences as tobacco counselors. Both moderators had received TTS training and were of Alaska Native heritage, which made it easier for them to quickly connect to participants through a mutual appreciation of core Alaska Native values and their TTS background.

Our approach has several strengths. The development of our training and content library was iterative and theory based. We used social media and health communication experts to conduct training grounded in situational learning. We used a combination of face-to-face and web-based formats and provided a scenario-based approach for moderators to practice their skills. All participants (moderators and trainers) in the training sessions served as experts to each other such that all participants were able to acquire new skills. We gathered feedback from the research team and our beta testers on how to improve the CAN Quit prototype. This feedback resulted in practical solutions that will be embedded in the RCT, which is beneficial for developing an intervention in phases (iteratively), as recommended by the National Institutes of Health and CDC guidelines [47,48]. Most importantly, our approach was created based on the need to improve use of evidence-based tobacco cessation resources specifically for Alaska Native people, a high-risk group for tobacco-related morbidity and mortality. This approach provided a systematic process to tailor or adapt content and training to appeal specifically to Alaska Native people living in Alaska.

Our approach also has some limitations. Implementing the prototype on Facebook results in a lack of control on some aspects of the intervention design, such as the order in which posts were introduced, which required moderator flexibility in responding to changes accordingly. Despite this limitation, given that Facebook is commonly used and familiar, less on-boarding of participants is required to participate in the intervention. In addition, a sample size of 10 was the minimum recommended for a group social media intervention; it is likely that engagement would be easier to achieve with a larger group size, as will be used for the RCT. Stimulating group interaction during the beta-testing phase presented a challenge to the moderators given the small number of participants and the short amount of time they participated. However, this challenge resulted in stronger engagement skills among moderators, which will aid in the RCT phase. Meanwhile, consistent with and building on the work of Pagoto et al [29], we came up with ten recommendations of lessons learned that could help guide others interested in developing similar social media intervention prototypes: (1) select moderators who can relate to your group; (2) build on existing skills of your moderators; (3) build community engagement; (4) use existing resources for content; (5) adapt content for cultural fit; (6) design content that highlights your goals; (7) include a wide variety of content and organize it into an easily searched library; (8) balance content with conversation; (9) support moderators to handle emotional communication; and (10) groom group participants as moderators. Table 3 outlines these recommendations in detail.

In summary, providing practical information on how we developed and evaluated our intervention prototype demonstrates how a beta-testing phase is essential in identifying how the platform, content, and moderation can be improved before rolling out a large-scale intervention. We learned that a social media group intervention is most likely to succeed if it balances well-developed culturally congruent content with strategies to engage Alaska Native participants in social media conversations facilitated by moderators. Moderators need to be provided with the necessary skills and methods for promoting web-based community engagement with and among group members and in encouraging behavior change. In our case, having Alaska Native moderators who were themselves Alaska Native and already experienced the challenges associated with smoking cessation was extremely helpful in both developing the training plan and promoting engagement. Future studies could explore using former intervention participants transitioning to a moderator role to enhance sustainability, growth, and feasibility of the intervention. Our next step will be to evaluate the prototype via a pilot RCT.

**Table 3:** Recommendations for creating a successful social media group to promote use of evidence-based smoking cessation resources for Alaska Native people.

Recommendation	Description
Select moderators who can relate to your group	We chose moderators with expertise in smoking cessation (TTS^a^) who also shared similar Alaska Native ancestry and cultural values. This approach is effective with building rapport among group participants and moderators.
Build upon existing skills of your moderators	Beyond training as TTS, our moderators received 20 additional hours of training in strategic web-based community management and health behavior change. We recommend training be scenario-based, experiential, and use actual posts for moderating practice.
Build community engagement	Our moderators shared that getting to know group participants made it easier to respond to their posts, reinforce quitting messages, and provide support. Train moderators to react, tag, and instant message participants to respond when they post. They should acknowledge active participants while also checking in with inactive participants, inviting them back to the group.
Use existing resources for content	We evaluated and adapted existing, evidence-based content already created by the CDC^b^ and ANTHC^c^, with their permission. This was both cost and time effective. We recommend using or refining existing high-quality content whenever possible, seeking necessary permissions.
Adapt content for cultural fit	We used an iterative theory-based process of qualitative and quantitative feedback from both participants and stakeholders to adapt our content and be responsive to the evolution of the participants and the group. We recommend using a conceptual framework with community and stakeholder input.
Design content that highlights your goals	Our goal was to promote use of evidence-based resources. We found it important to include links to the Alaska Quitline or Tribal cessation resources on all content. We suggest including a call to action with each post.
Include a wide variety of content and organize it into an easily searched library	It is difficult to predict how participants will communicate and what will motivate them to engage in the group. Having an organized library with a variety of content allows moderators flexibility to select content that is responsive to an ongoing conversation or that meets the needs or interests of group participants to maintain engagement.
Balance content with conversation	We used content that fit concerns and conversations happening within the group. We believe the key to success lies in fostering group engagement through conversation with moderators and with each other. It is necessary to create a community of talkers, not readers.
Support moderators to handle emotional communication	We provided 20 hours of MI^d^ and web-based community management training to prepare moderators to support group participants who share sensitive information. We recommend training also include a plan for moderators to seek additional support.
Groom group participants to be moderators	Once our group was underway, we saw some participants were very engaged, not only with moderators but also with other group participants. Consider inviting and training former group participants to volunteer as peer mentors or moderators.

^a^TTS: trained tobacco specialist.

^b^CDC: Centers for Disease Control and Prevention.

^c^ANTHC: Alaska Native Tribal Health Consortium.

^d^MI: motivational interviewing.

## Abbreviations

ANTHC: Alaska Native Tribal Health Consortium
CAN Quit: Connecting Alaska Native People to Quit Smoking
CDC: Centers for Disease Control and Prevention
MI: motivational interviewing
RCT: randomized controlled trial
TTS: trained tobacco specialist
